# Vapors Produced by Electronic Cigarettes and E-Juices with Flavorings Induce Toxicity, Oxidative Stress, and Inflammatory Response in Lung Epithelial Cells and in Mouse Lung

**DOI:** 10.1371/journal.pone.0116732

**Published:** 2015-02-06

**Authors:** Chad A. Lerner, Isaac K. Sundar, Hongwei Yao, Janice Gerloff, Deborah J. Ossip, Scott McIntosh, Risa Robinson, Irfan Rahman

**Affiliations:** 1 Department of Environmental Medicine, University of Rochester Medical Center, Rochester, NY, United States of America; 2 Department of Public Health Sciences, University of Rochester Medical Center, Rochester, NY, United States of America; 3 Mechanical Engineering Department, Rochester Institute of Technology, Rochester, NY, United States of America; University of Texas Medical Branch, UNITED STATES

## Abstract

Oxidative stress and inflammatory response are the key events in the pathogenesis of chronic airway diseases. The consumption of electronic cigarettes (e-cigs) with a variety of e-liquids/e-juices is alarmingly increasing without the unrealized potential harmful health effects. We hypothesized that electronic nicotine delivery systems (ENDS)/e-cigs pose health concerns due to oxidative toxicity and inflammatory response in lung cells exposed to their aerosols. The aerosols produced by vaporizing ENDS e-liquids exhibit oxidant reactivity suggesting oxidants or reactive oxygen species (OX/ROS) may be inhaled directly into the lung during a “vaping” session. These OX/ROS are generated through activation of the heating element which is affected by heating element status (new versus used), and occurs during the process of e-liquid vaporization. Unvaporized e-liquids were oxidative in a manner dependent on flavor additives, while flavors containing sweet or fruit flavors were stronger oxidizers than tobacco flavors. In light of OX/ROS generated in ENDS e-liquids and aerosols, the effects of ENDS aerosols on tissues and cells of the lung were measured. Exposure of human airway epithelial cells (H292) in an air-liquid interface to ENDS aerosols from a popular device resulted in increased secretion of inflammatory cytokines, such as IL-6 and IL-8. Furthermore, human lung fibroblasts exhibited stress and morphological change in response to treatment with ENDS/e-liquids. These cells also secrete increased IL-8 in response to a cinnamon flavored e-liquid and are susceptible to loss of cell viability by ENDS e-liquids. Finally, exposure of wild type C57BL/6J mice to aerosols produced from a popular e-cig increase pro-inflammatory cytokines and diminished lung glutathione levels which are critical in maintaining cellular redox balance. Thus, exposure to e-cig aerosols/juices incurs measurable oxidative and inflammatory responses in lung cells and tissues that could lead to unrealized health consequences.

## Introduction

The consumption of electronic nicotine delivery systems (ENDS) and electronic cigarettes (e-cigs) is rising and currently scientific information necessary to inform the FDA and clinicians of potential health risks is lacking. Studies involving the effects of ENDS/e-cig liquids and aerosols on animal cells and tissues, in particular those of the lung, are lacking and the long-term outcome of chronic ENDS use is difficult to predict. Oxidative toxicity and inflammation are associated with increased risk of lung diseases caused by conventional tobacco products is well established [1]. However, there is no clear indication that inhaling aerosols from ENDS/e-cigs (as a cessation device) will allow a healthy outcome for users and furthermore, the manufactures that produce ENDS globally are not liable to disclose the materials and chemicals employed in their fabrication.

Two independent studies have reported that certain flavored e-liquids exhibit differential *in vitro* cytotoxicity when applied directly to various cells independent of nicotine, suggesting potential toxicities are associated with flavor additives [2,3]. Other toxic chemicals including carcinogens which are not typically found in e-liquids may be released or generated from ENDS/e-cigs and have been detected at low levels in various ENDS aerosols [4–6]. Some of these toxicants may emanate from heated structural materials while drawing air through an ENDS device, but are also proposed to form during the vaporization process [7,8]. Specific particulates, heavy metals, and toxic carbonyls in ENDS/e-cig aerosols have recently been measured in e-cigs aerosols as well [5,7,9,10].

Despite limited evidence that ENDS/e-cigs pose a danger, there is debate as to whether meaningful comparisons exist between the health risks of those exposed to tobacco smoke and those exposed to aerosols generated by ENDS devices [11]. Many of the secondary compounds (polyaromatic hydrocarbons, PAHs, aldehydes, and carbonyls) identified in ENDS aerosols and replacement liquids (e-liquids) are considered low level, especially in comparison to levels measured in environmental tobacco/cigarette smoke [5–7,12,13]. Furthermore, the levels of toxic compounds identified in ENDS aerosols that primary users would be exposed to in a “vaping” session are also not expected to approach established threshold limit values for what is considered a health risk for by-standard exposure to these compounds in cigarette smoke (passive smoking/second hand smoke) [14]. However, oxidants/reactive oxygen species (OX/ROS) found in cigarette smoke and generated from tars are major contributors in mediating an inflammatory state, which have been implicated in the pathogenesis of diseases, such as chronic obstructive pulmonary disease (COPD) and lung cancer [15]. The presence or generation of OX/ROS associated with ENDS devices and e-liquids has yet to be evaluated and may pose a health risk that is underappreciated.

There are approximately 10^15^ free radicals in a puff of conventional cigarette smoke in addition to heavy metals nanoparticles which have also recently been shown in e-cig aerosols to similar levels per “puff” [10,16]. Heavy metals may undergo redox cycling and alter the oxidation state of the cell by potentiating the production of ROS [17]. It is expected that OX/ROS in aerosols of ENDS/e-cigs will have an impact on cellular oxidative stress, redox imbalance, and lung inflammation, but this is still not clear *in vitro* in lung cells and *in vivo* in lungs. We hypothesized that electronic nicotine delivery systems (ENDS)/e-cigs induce oxidative toxicity and inflammatory response by generation of ROS and alteration in redox GSH levels in lung cells *in vitro* and *in vivo* in mouse lung exposed to their aerosols, respectively.

We determined the source of oxidants produced from ENDS/e-cigs by a modified 2'-7'-dichlorodihydrofluorescein diacetate (DCFH-DA) fluorescein derived dye to detect OX/ROS reactivity in ENDS/e-cig aerosols and pre-vaporized e-liquids in a cell free system. We also evaluated cultured lung cells exposed to e-liquids or aerosols for cell toxicity, inflammation, and then extended our studies to a mouse model of e-cig aerosol exposure. By exposing wild-type (C57BL/6J) mice to e-cig aerosols, we examined the effect of short-term (3 days) exposure to e-cig aerosols on aspects of lung inflammation, oxidative stress, and redox physiology by measuring changes in glutathione levels.

## Materials and Methods

### Ethics statement

All experimental protocols were performed in accordance with the standards established by the United States Animal Welfare Act, as set forth by the National Institutes of Health guidelines. The research protocol for these studies was approved by the University of Rochester Committee on Animal Research.

### Materials

**Fig 1:**
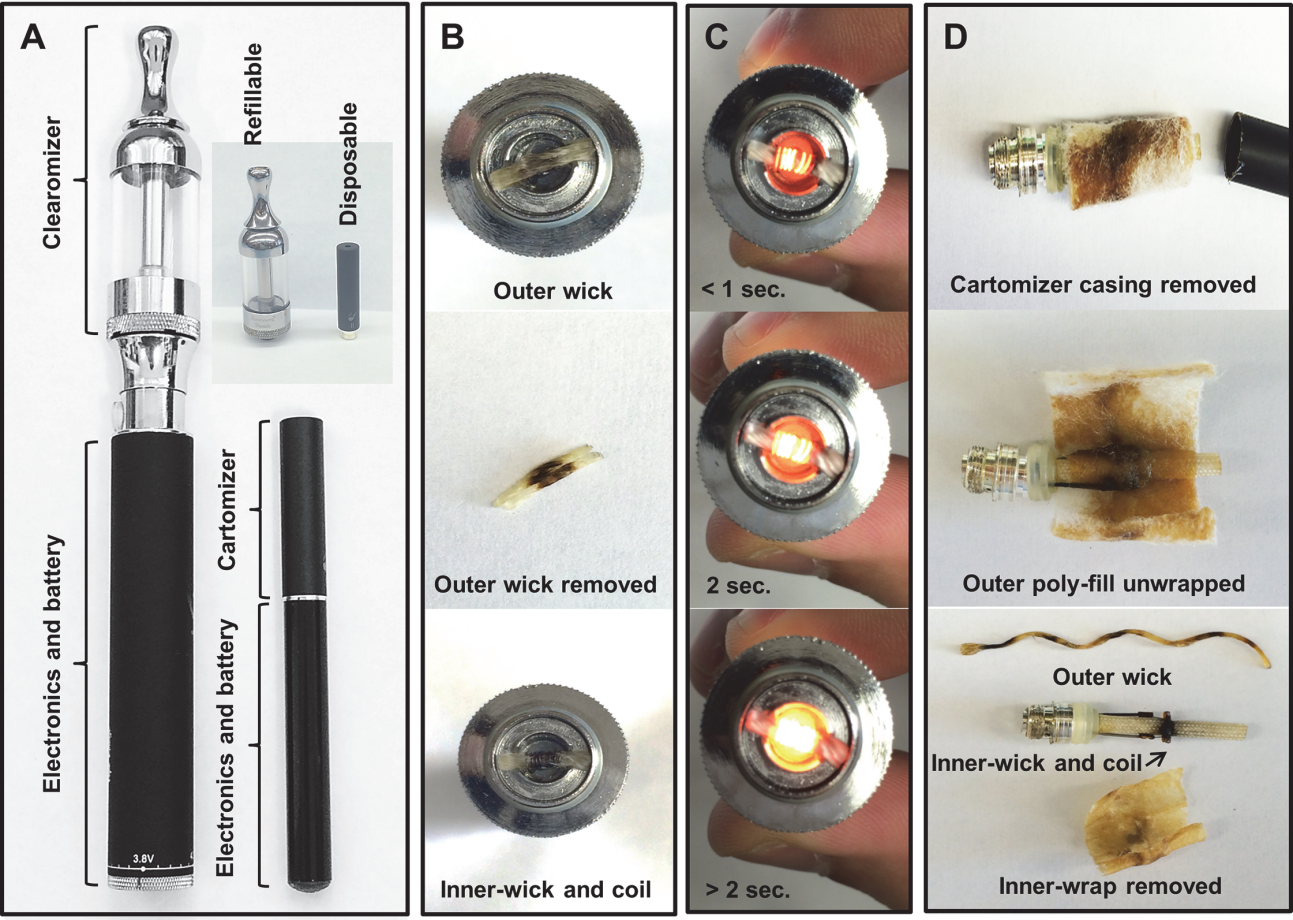
Refillable ENDS and Blu e-cigarettes. (A) Refillable ENDS and Blu e-cigarettes (B) Clearomizer removed, outer wick shown covering top of heating element coil (Upper panel). Used wick removed showing darkened region that contacted heating coil (Middle panel). Heating coil wrapped around second wick (Lower panel). (C) Activating heating element on refillable ENDS with Clearomizer removed. Less than 1 second activation (Upper panel), 2 seconds activation (Middle panel), greater than 2 second’s activation (Lower panel). (D) Cartomizer casing removed after previous use (Upper panel). Poly-fill material with partially absorbed e-liquid wrapped around the core. Outer material removed exposing inner absorbent material tightly wrapped around heating element (Middle panel). Heating element exposed showing coil wrapped wick secured perpendicular to longer woven polymer tubing. A long thin fiber that was wrapped around the heating coil shows points of contact with coil wire (Lower panel).

Two ENDS devices were used. First, a refillable pen style ENDS (eGo Vision Spinner battery, China) and compatible clearomizer chamber (Anyvape, China) with 2.2 ohm heating element was purchased from local retailers (**Fig. 1A**). The clearomizer chamber can be easily filled with e-liquid of choice allowing liquid to continuously absorb into heating element wick. Refillable e-liquids (**Table 1)** for use with refillable ENDS were purchased from various local retailers. Second, is the Blu e-cigs where the cartomizer is manufactured to be disposable when the pre-loaded e-liquid is exhausted. The Blu e-cigs and disposable cartomizer cartridges were purchased from local retailers (**Fig. 1A**).

**Table 1:** ENDS e-liquids their trade name, flavor, and manufacturer information obtained from local retailers used in this study.

Trade Name	Flavor	Form	Manufacturer
Blu	Classic Tobacco	Cartomizer	Lorillard
Blu	Magnificent Menthol	Cartomizer	Lorillard
Drip	Berry Intense	e-Liquid	Vaporotics
Drip	Melon Mania	e-Liquid	Vaporotics
Drip	Peaches ‘n Cream	e-Liquid	Vaporotics
Drip	Pineapple Express	e-Liquid	Vaporotics
Ecto	American Tobacco	e-Liquid	Ecto
Encore	Tobacco	e-Liquid	Encore Vapor Inc.
Roc Juice	Tobacco	e-Liquid	Roc Juice Inc.
Roc Juice	Coconut	e-Liquid	Roc Juice Inc.
Upstate Vape	Mountain Dew	e-Liquid	Upstate Vape
Upstate Vape	Marbo	e-Liquid	Upstate Vape
Upstate Vape	9x Tobacco	e-Liquid	Upstate Vape
Vapor Drops	AMP	e-Liquid	-
Vapor Drops	Very Berry	e-Liquid	-
Vapor Drops	Tobacco	e-Liquid	-
Vape Dudes	Cinnamon Roll	e-Liquid	Vape Dudes
Vape Dudes	Classic Tobacco	e-Liquid	Vape Dudes
Vape Dudes	Cotton Candy	e-Liquid	Vape Dudes
Vape Dudes	Grape Vape	e-Liquid	Vape Dudes
Vape Dudes	Strawberry Fields	e-Liquid	Vape Dudes
Vape Dudes	Strawberry Zing	e-Liquid	Vape Dudes

### Cell-free ROS assay

The relative levels of OX/ROS produced from e-cig vapor or smoke (Federal Trade Commission protocol) using a CSM-SSM machine (CH-Technologies Inc.) from filtered research grade cigarettes (3R4F) was determined using 2’,7’dichlorofluorescein diacetate (H2 DCF-DA) fluorogenic probe (EMD Bioscience, CA) as described previously [18,19]. In brief, aerosols generated from ENDS/e-cigs were tested for OX/ROS passing through tubing over a distance similar to that from the mouth to the bifurcation of the human trachea (approximately 20–24 cm) [20]. For each exposure, 5 ml of dichlorofluorescein DCFH-HRP solution [21,22], was loaded into a clean glass bubbler (Prism research). A lab pump (FMI, Syosset, NY) with a flow range of 0-1296 ml/min was switch activated using an FMI stroke rate controller set at 60% flow to draw a steady stream of e-cig aerosols/cigarette smoke directly through the DCFH solution. E-cig aerosols were pulsed through DCFH in the bubbler at room temperature for 4–5 seconds [23] at 30 second intervals for a total of 10 minutes. Following exposures, sample tubes were placed on ice and protected from light sources until analysis. A spectrofluorometer (Turner Quantech fluorometer Model FM109535 from Barnstead International/Thermolyne Corporation) was used to measure oxidized dichlorofluorescein (DCF) fluorescence at absorbance/emission maxima of 485 nm/535 nm. Hydrogen peroxide standards between 0 and 50 μM were created from 1 M stock and reacted at room temperature for 10 minutes with prepared DCFH solution in a total of 5 ml. These standards were then used to calibrate fluorescence intensity units (FIU) which numerically match respective hydrogen peroxide (H_2_O_2_) concentrations. The DCF fluorescence data are expressed as μM H_2_O_2_ equivalents referring to the concentration of the H_2_O_2_ added to the DCFH solution [16,19,24].

### Cell culture and treatments

Human bronchial airway epithelial cells (H292) and human fetal lung fibroblasts (HFL1) were obtained from American Type Culture Collection (Manassas, VA). H292 cells were cultured in RPMI-1640 supplemented with 10% FBS, 2 mM L-glutamine, 100 μg/ml penicillin and 100 U/ml streptomycin. HFL1 cells were cultured in DMEM-F12 supplemented with 10% FBS, 50 μg/ml penicillin, and 50 U/ml streptomycin. Human bronchial epithelial cells (Beas-2B) were grown in DMEM-F12 supplemented with 5% FBS, 15 mM HEPES, 100 μg/ml penicillin, and 100 U/ml streptomycin. The cells were grown at 37°C in a humidified atmosphere containing 5% CO_2_ and 3% O_2_ controlled incubator. HFL-1 cells were grown to 80–90% confluence and replaced with 0.5% FBS 24 hrs prior to treatment. HFL-1 was treated with the following e-liquids: Propylene glycol, Glycerin, Vape Dudes (Classic tobacco with or without nicotine), Vape Dudes (Cinnamon roll without nicotine), Vape Dudes (Grape vape without nicotine), Ecto (American tobacco with or without nicotine) and other e-liquids (**Table 1**) for 24 hrs and then examined for morphological changes by phase-contrast microscopy at 20x magnification.

### Preparation of aqueous cigarette smoke extract

Research grade cigarettes 3R4F were obtained from the Kentucky Tobacco Research and Development Center at the University of Kentucky (Lexington, KY). Cigarette smoke extract (CSE) was prepared by bubbling smoke from one cigarette into 10 ml serum-free media at a rate of one cigarette/min as described previously [25–27]. The CSE solution was then filter sterilized with a 0.45 μm syringe filter. CSE preparation was standardized by measuring the absorbance (OD: 1.00 ± 0.05) at a wavelength of 320 nm. The pattern of absorbance (spectrogram) observed at 320 nm showed very little variation between different preparations of CSE. CSE was freshly prepared for each experiment and diluted with culture media supplemented with 10% FBS immediately before use. For CSE treatments, HFL-1 cells were grown to 80–90% confluence and replaced with 0.5% FBS in DMEM (supplemented with 50 μg/ml penicillin, and 50 U/ml streptomycin) 24 hrs prior to CSE treatment at 37°C in a humidified atmosphere containing 5% CO_2_ and 3% O_2_ controlled incubator.

### Air-liquid interface cell culture and exposure

H292 lung epithelial cells (ATCC) were grown at 37°C in a humidified atmosphere containing 5% CO_2_, and 3% O_2_ controlled incubator to 80–90% confluence in RPMI-1640 supplemented with 10% FBS, 2 mM L-glutamine, 100 U/ml penicillin, and 100 μg/ml. streptomycin. Human bronchial epithelial cells (Beas-2B) were grown in DMEM-F12 supplemented with 5% FBS, 15 mM HEPES, 100 μg/ml penicillin, and 100 U/ml streptomycin. Cells were then sub-cultured into porous transwells (0.4 micron, Corning, Corning NY) at a density of 200,000 cells/transwell in 6-well plates. Transwell cultures were then placed into air-liquid interface exposure chamber [28–31]. Before placement into exposure chamber, cells were minimally overlaid with approximately 200 μl of RPMI media to prevent drying. Media is continuously exchanged through the sealed chambers ports via peristaltic pump while in contact with the porous bottom of the transwell. Using tubing connected to a lab pump (FMI, Syosset, NY). Blu e-cig aerosol (Classic tobacco flavor containing 16 mg nicotine) using a CSM-SSM machine (CH-Technologies Inc.) was drawn into the chamber every 30 seconds with a 4 second pulse [23] for different time durations 5, 10, and 15 minutes respectively. No treatment control/air group H292 cells were maintained at 37°C in a humidified atmosphere containing 5% CO_2_, and 3% O_2_ controlled incubator for 16 hrs after Blu e-cig aerosol exposure to condition media.

### Cell viability and flow cytometry

HFL-1 cells were cultured at 37°C, in a humidified atmosphere containing 5% CO_2_, and 3% O_2_ controlled incubator either in a 24-well plates (2.0 cm^2^) or 6-well plates (9.6 cm^2^) using appropriate growth media as described earlier. When the cells were about 80–90% confluent, fresh media was replaced with 0.5% FBS in DMEM (supplemented with 50 mg/ml penicillin, and 50 U/ml streptomycin) with or without e-liquids or CSE. Cell viability was measured after 24 hrs treatment by acridine orange/propidium iodide (AO/PI) staining using Cellometer 2000 (Nexcelom Bioscience, Lawrence MA). Beas-2B cells were trypsinized following 15 min exposure to Blu e-cig vapor in air-liquid interface chamber as described earlier. Following e-cig aerosol exposure, cells were washed in PBS, and analyzed on BD-LSRII system. An increase in cellular fluorescence after e-cig aerosol exposure was detected using a Violet B 405 nm laser and 440/40 band-pass filter. Data was compiled on FlowJo V. 10.

### E-cigarette aerosol mouse exposure

Eight weeks old C57BL/6J mice were housed in the Inhalation Core Facility at the University of Rochester before being exposed to room air or e-cig aerosol exposure which was adapted as described previously for conventional cigarettes [32,33]. Blu e-cig (Classic tobacco flavor containing 16 mg nicotine) were used to generate the aerosols by a Teague smoking machine (Model TE-10, Teague Enterprises, Woodland, CA) at a concentration of approximately 200 mg/m^3^ TPM. Mice received 5 h exposures per day for 3 successive days. A new TE-10 Teague machine was modified and dedicated only for ENDS/e-cig aerosol exposures *in vivo*. All animal protocols described in this study were approved by the University Committee on Animal Research Committee of the University of Rochester.

### Bronchoalveolar lavage

Mice were anesthetized by an intraperitoneal injection of pentobarbital sodium (100 mg/kg; Abbott Laboratories, Abbott Park, IL) and then sacrificed by exsanguination in two different batches one immediately after 5 hrs e-cig exposure on the 3^rd^ day and another batch 24 hrs after last e-cig exposure. The lungs were lavaged three times with 0.6 ml of saline via a cannula inserted into the trachea. The aliquots were combined and centrifuged, and the bronchoalveolar lavage (BAL) fluid stored at -80°C for cytokine/chemokine analysis and the cell pellet was resuspended in saline. The cells were stained with AO/PI stain and the total cell number was counted using Cellometer 2000 (Nexcelom Bioscience, Lawrence MA). Cytospin slides (Thermo Shandon, Pittsburgh, PA) were prepared using 50,000 cells per slide, and differential cell counts (~500 cells/slide) were performed on cytospin-prepared slides stained with Diff-Quik (Siemens, DE).

### Pro-inflammatory mediators analysis

Following 24 hrs humectant/e-liquid treatment, conditioned media was collected and stored at -80°C for measuring pro-inflammatory mediators. IL-8 and IL-6 levels were measured by enzyme-linked immunosorbent assay (ELISA) according to manufacturer’s instructions (Life Technologies, Carlsbad, CA). Pro-inflammatory mediators in bronchoalveloar lavage fluid (BALF) collected from room air and e-cig aerosol exposed mice 24 hrs after the last Blu e-cig exposure were measured using ELISA according to the manufacturer’s instructions (MCP-1 and IL-6). Various cytokines/chemokines from BAL fluid were measured by the Luminex Flexmap3D system (Austin, TX) using Milliplex mouse cytokine/chemokine magnetic bead panel for Luminex platform according to manufacturer’s instructions (Billerica, MA).

### Cotinine assay

Levels of cotinine in mouse plasma samples collected immediately after the 3^rd^ day Blu e-cig aerosol exposure (5 hrs) was measured by ELISA according to manufacturer’s instructions (Abnova, Taipei, TW).

### Glutathione and glutathione disulfide measurements

Total and oxidized (disulfide) glutathione levels measured in mouse lung harvested immediately after the 5 hrs Blu e-cig aerosol exposure (3^rd^ day) as described previously [34]. In brief, the concentration of total glutathione in the supernatant of lung homogenates was determined by comparison with the colorimetric rate of DTNB reduction by known standard concentrations of reduced glutathione (GSH). For determining the concentration of oxidized glutathione/glutathione disulfide (GSSG), lung homogenates were combined with 2% of 2-vinylpyridine (VP) to derivatize (masking) endogenous GSH. Excess VP is neutralized by triethanolamine so that in the subsequent reaction, glutathione reductase is able to recycle endogenous GSSG back into underivatized GSH. GSSG levels are then indirectly measured by DTNB reduction by newly reduced GSH, which was produced from endogenous GSSG *in vitro*. Results were expressed as the nmol of total glutathione and GSSG per mg protein as well as total glutathione/GSSG ratio. All sample homogenates were prepared using RIPA buffer and underwent multiple freeze thaw cycles prior to measuring glutathione levels.

### Statistical analysis

Statistical analysis of significance was calculated using unpaired Student’s *t*-test. Probability of significance compared to control was based on 2-tail *t*-tests and indicated in figure legends. The results are shown as the mean ± SD unless otherwise indicated. A value of *P* < 0.05 is considered as statistically significant.

## Results

### ENDS/e-cigarette OX/ROS generation

OX/ROS produced by ENDS/e-cigs were detected by drawing the aerosols through a fluorescein derived dye (DCFH solution) using an air flow pump (see Materials and Methods). The oxidized form of DCFH (DCF) emits green fluorescence following excitation at 490 nm indicating OX/ROS or ROS activity. In both cell and cell-free systems, DCFH serves as a semi-quantitative indicator for presence of reactive OX/ROS and has been used previously to measure nanoparticle mediated oxidation in cell free systems [35].

**Fig 2:**
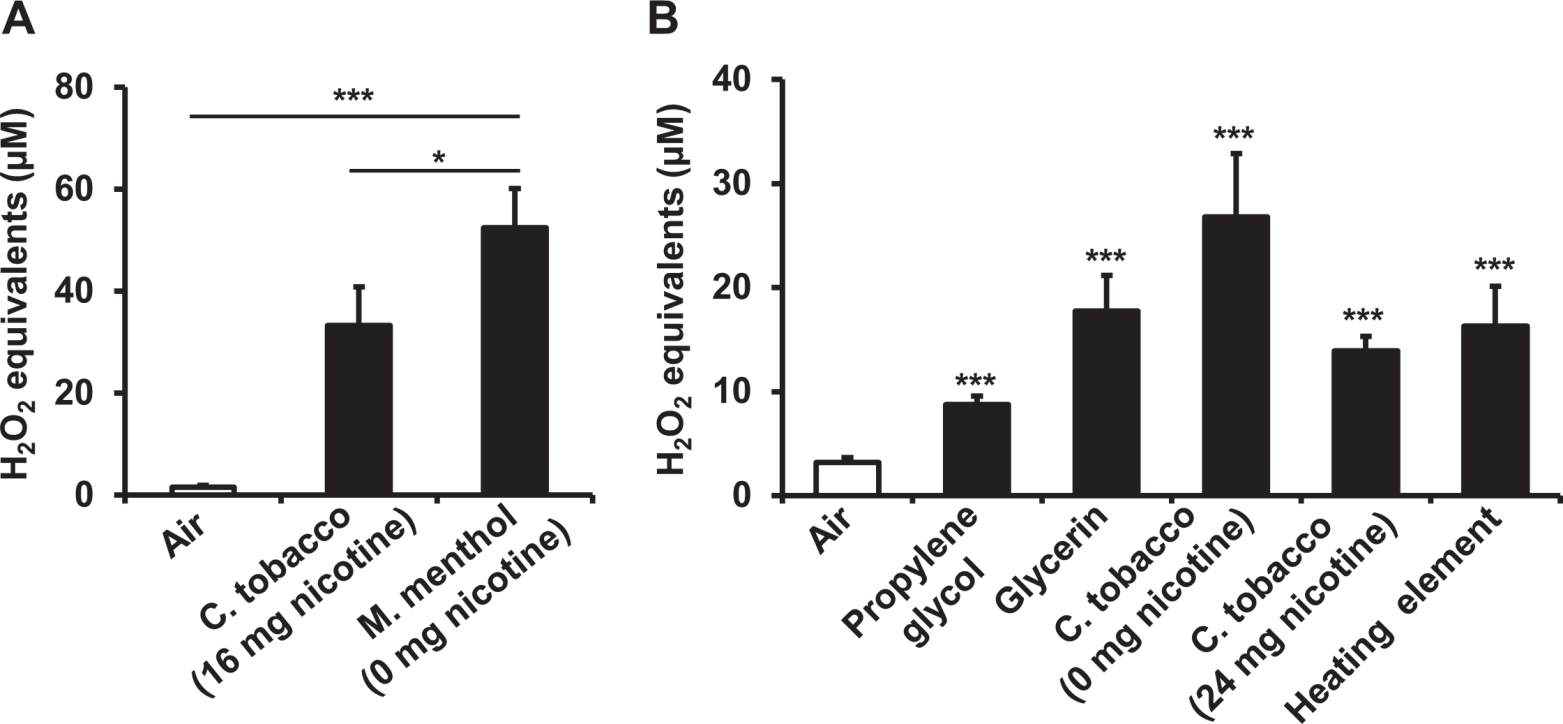
OX/ROS in ENDS vapor. Aerosols or air-sham control drawn through DCFH OX/ROS indicator solution. (A) Blu e-cigarette cartomizers; Classic tobacco or Magnificent menthol flavor e-cigs. Data are shown as mean ± SD (n = 3/group).* P < 0.05, *** P < 0.001 compared to air-sham control (B) eGo refillable vaporizer. Humectants; propylene glycol and glycerin. Commercial e-liquid refills; Vape Dudes Classic tobacco flavor. Data are shown as mean ± SEM (air, n = 15; propylene glycol, n = 23; glycerin, n = 21; Vape Dudes C. tobacco 0 mg nicotine, n = 7; Vape Dudes C. tobacco 24 mg nicotine, n = 3; Heating element, n = 9). *** P < 0.001 compared to air-sham control.

Detection for the presence of OX/ROS in Blu e-cig vapor was performed using two different flavored Blu e-cig cartomizers (Classic Tobacco or Magnificent Menthol) (**Fig. 1A)**. Each cartomizer varies in nicotine content (0 mg and 24 mg) and both were included to assess if aerosols produced within the cartomizers give rise to major differences in DCF fluorescence intensity after they were drawn through DCFH solution. Aerosols drawn through DCFH produced from the classic tobacco flavor cartomizer (16 mg of nicotine) resulted in increased H_2_O_2_ μM equivalents (equivalent to DCF fluorescence intensity units) as compared to air-sham group (**Fig. 2A**). The levels of H_2_O_2_ μM equivalents from the menthol cartomizer aerosols were also significantly increased (**Fig. 2A**). Comparison of OX/ROS levels between both of the cartomizer aerosols showed that the one containing nicotine resulted in significantly reduced levels of H_2_O_2_ μM equivalents (**Fig. 2A**).

Next, we exchanged the Blu e-cigs with a different type of popular refillable ENDS to test for OX/ROS reactivity in ENDS aerosol. The eGO Vision Spinner with a 2.2 ohm “wicked” heating element and clearomizer chamber capable of holding 4.5 ml of e-liquid is noticeably larger than the Blu e-cig (**Fig. 1A**) and produces aerosols in a similar fashion. The e-liquids, sold in a plethora of flavors are primarily comprised of humectants propylene glycol and glycerin [36]. Propylene glycol was filled into the clearomizer and aerosols produced from the refillable ENDS elicited an increase in H_2_O_2_ μM equivalents as compared to air-sham group (**Fig. 2B).** Similarly, aerosols produced exclusively from glycerin also reacted with DCFH leading to significantly increased H_2_O_2_ μM equivalent levels (**Fig. 2B**). Two of the commercially available e-liquids (Vape Dudes and Classic tobacco flavor) were also tested for OX/ROS reactivity using the refillable ENDS device. One of these samples contains 0 mg nicotine and the other contains 24 mg nicotine in addition to undisclosed mixtures of propylene glycol, glycerin, and flavor additives. Both the non-nicotine and nicotine containing commercially available e-liquids produced aerosols that resulted in increased H_2_O_2_ μM equivalents levels (**Fig. 2B**).

These results suggest OX/ROS are emanating from the e-cigs/e-liquids and are associated with the aerosols that are drawn through the DCFH indicator, and nicotine was not likely a sole contributing factor in increased OX/ROS reactivity.

### Source of OX/ROS generation from ENDS/e-cigarettes

It was not clear if the OX/ROS we detected were exclusive to the aerosols of the ENDS or if they might emanate from another source within the device. We determined that there are two possible sources of OX/ROS that are generated by the refillable ENDS device. One of the sources of OX/ROS appears to be the heating element since there is an increase in OX/ROS when the heating element is activated without e-liquid filled into the clearomizer chamber (**Fig. 2B**). In this case, air is drawn through the device by the pump as the ENDS device is activated, however, there was no visible sign of aerosol being produced. From this data, we conclude it is possible to generate OX/ROS from ENDS independent of e-liquid vaporization.

OX/ROS detection in ENDS aerosols overall yielded a rather broad range of measurements for DCF fluorescence including what we defined as “high range” values. Attaining a high range value measurement required a 1:10 dilution in pristine DCFH solution to extrapolate their final values. High range aerosol-DCF fluorescence values, including the values for OX/ROS detected in ambient air flow from activating the heating element without e-liquids, were partitioned and compiled together (**Table 2**).

**Table 2:** DCF fluorescence values obtained for refillable ENDS aerosols or ambient air alone drawn through DCFH in cell-free ROS assay.

Humectants	**H**_**2**_ **O**_**2**_ **equivalents (μM)** ^†^
Propylene glycol	120.7
	129.0
	127.3
Mean ± SEM ^‡^	125.7 ± 2.5*** (93.0) ^#^
Glycerin	211.5
	305.2
	312.4
	360.7
	200.2
	145.6
Mean ± SEM ^‡^	255.9 ± 33.6*** (93.1) ^#^
Propylene glycol: Glycerin (50:50)	412.5
	360.5
	146.8
Mean ± SEM ^‡^	306.6 ± 81.3*** (97.0) ^#^
Classic tobacco (0 mg nicotine)	248
	113.6
	103.2
	134.0
	131.0
Mean ± SEM ^‡^	146.0 ± 26.1*** (81.6) ^#^
Classic tobacco (24 mg nicotine)	327.7
	103.8
	60.4
Mean ± SEM ^‡^	164.0 ± 82.8*** (91.5) ^#^
Pre-used heating element (without e-liquid)	250.5
	192.0
	84.2
Mean ± SEM ^‡^	175.6 ± 48.7*** (90.7) ^#^
Air-Sham (control)	3.89
	4.6
	4.0
	2.1
	1.5
	1.6
	1.4
	1.2
	1.2
	2.5
	4.6
	2.8
	4.8
	4.9
	7.1
Mean ± SEM ^‡^	3.2 ± 0.46

DCF fluorescence values (high range) from refillable ENDS aerosols or ambient air flowing through activated ENDS heating element. Each fluorometer reading indicates that oxidation to DCF is diluted 1 to 10 with pristine DCFH solution to attain fluorometer measurements within calibration range of the high standard (50 μM H_2_O_2_).

† Each value shown in H_2_O_2_ equivalents (μM) for humectants represents individual trials analyzed by cell-free ROS assay.

‡ Compared with Air-Sham (control) and from values shown in Fig. 2B.

♯ The percentage of change from non-high range to high range values is based on values obtained from data quantitated in Fig. 2B and compared to the high range values in Table 2.

***P<0.001 vs Air-Sham (control) and from values shown in Fig. 2B.

To validate whether or not OX/ROS reactivity emanating from the refillable ENDS device occurs either by vaporizing e-liquids/humectants, or activating the heating element without e-liquids/humectants, we hypothesized that the state of the heating element (new versus multi-use) affects the capacity for OX/ROS to be generated by the refillable ENDS. We first cleaned and refilled the removable clearomizer chambers with 2.0 ml of either propylene glycol, glycerin, or a commercial refill e-liquid (Vape Dudes Classic tobacco, 0 mg nicotine). A new set of new replacement 2.2 ohm heating elements was obtained from a local merchant that sells e-cigs and accessories and three of the heating elements that were used a number of times in previous experiments (over 50 times use, exact number of uses unknown) retained. For each e-liquid/humectant, two repeat trials were conducted with a pre-used heating element, drawing aerosols into DCFH solution in exactly the same manner and timing as for our previous DCFH experiments. Aerosols for each e-liquid/humectant drawn through DCFH indicate the presence of OX/ROS compared to air-sham control which did not result in any appreciable level of OX/ROS reactivity (**Table 3, Experiment 1**). Next, in order to determine if replacing the pre-used heating element with a new one is able to achieve “high range” range DCF fluorescence, the same sample of e-liquid/humectant in the clearomizer from Trial 1 and Trial 2 was retained. Each DCF fluorescence value obtained after installing the new heating element required 1:10 dilutions in DCFH solution (**Table 3, Experiment 1**). These results suggest that the state of heating element after activation affects the generation of OX/ROS by the refillable ENDS.

**Table 3:** State of the refillable ENDS heating element and its influence over successive use to generate OX/ROS in a cell-free ROS assay.

	State of the heating element			
***Experiment 1***	**Pre-used**		**New**	
**Humectants**	**Trial 1**	**Trial 2**	**Single use only**	
Propylene glycol	15.32	13.06	127.23	
Glycerin	20.65	34.97	305.2	
Consumer refill	47.55	37.42	133.97	
Air (sham)	1.19	2.08	1.17	

***Experiment 2***	**New**	**2^nd^ use**	**3^rd^ use**	**4^th^ use**
Powered	33.28	8.99	5.68	135.6†
Air (sham)	1.60	1.50	1.39	-

***Experiment 3***	**Pre-used**			
	**Clearomizer filled with e-liquid**		**Emptied clearomizer with wicked e-liquid**	
**Humectant**	**Trial 1**	**Trail 2**	**Trial 3**	**Trial 4**
Consumer refill	47.55	37.42	192.40	250.50

Each flourometric value shown in this table represents the H_2_O_2_ equivalents (μM) measured after aerosols produced from different humectants/Vape dudes e-liquids (classic tobacco, 0 mg nicotine) or ambient air moving through the activated device are drawn through DCFH solution to test the role of heating element state by cell-free ROS assay. DCF fluorescence values less than 3.2 H_2_O_2_ equivalents (Average air-sham control values determined in **Fig. 2B)** were not considered to contain oxidants.

† Fluorometric value shown in 4th use is after direct addition of e-liquid to the wick analyzed by cell-free ROS assay.

*Experiment 1*: Clearomizer chamber is filled with ~2.0 mL humectant or e-liquid. A previously used heating element is installed into the device for Trial 1 and 2. The third trial is carried out after exchanging the used heating elements for new ones (single use).

*Experiment 2*: A never before used heating element is installed into the refillable ENDS. The ENDS is activated and ambient air is drawn through the device and then into DCFH solution. The experiment is repeated for successive 3 trials using that same heating element. After the third trial (heating element 3^rd^ use), 2 drops of e-fluid is “dripped” onto the wick and allowed to absorb. Aerosols are then produced from the “dripped” e-liquid and drawn into DCFH (4^th^ use).

*Experiment 3*: After loading the clearomizer with ~ 2.0 of e-liquid, a heating element used from previous experiments is installed into the device and the e-liquid aerosols that it produces are drawn into DCFH for 2 trials. For trials 3 and 4, the clearomizer chamber is completely emptied and the used heating element wick allowed to retain absorbed e-liquid before producing aerosols and drawing them into DCFH.

We further confirmed that the state of the heating element affects OX/ROS generation by installing a new heating element and activating it independently of e-liquids (empty clearomizer) for three trials. As the state of the heating element transitions from new to multi-used between trials, it’s generation of OX/ROS approached air-sham control level of DCF fluorescence (**Table 3, Experiment 2, New, 2** ^**nd**^ **use, and 3** ^**rd**^ **use)**. We then hypothesized that if the vaporization process of the e-liquids is also a source of OX/ROS generation, then adding e-liquid for the 4^th^ use of the used heating element will lead to a spike in DCF fluorescence after the aerosols are drawn through DCFH solution. Rather than filling the clearomizer with e-liquid, a single drop of e-fluid was absorbed into the heating element wick. The 4^th^ use heating element trial was completed after 5 minutes (half number of puffs) rather than the usual 10 minutes for all other trials. The resultant DCF fluorescence value required a 1:10 dilution in DCFH solution (**Table 3, Experiment 2, 4** ^**th**^ **use)**. Overall, these results suggest that there are at least two possible sources of OX/ROS released from ENDS, 1) from activation of the heating element, and 2) the process of vaporizing e-liquids.

### ENDS “dripping” technique and OX/ROS generation

The use of a refillable clearomizer chamber for ENDS is typical for securing e-liquids while consumers inhale their aerosols. An emerging trend abandons use of the clearomizer and replaces it for an inhalation tip that does not hold e-fluid. The “drip tip” allow consumers to “drip” e-liquid directly onto the heating element wick in the same manner as we applied e-liquid to the heating element for the 4^th^ use (**Table 3, Experiment 2**). To determine whether or not the clearomizer filled with e-liquid versus dripping the e-liquid onto the heating element wick leads to high range fluorescence values (requires 1:10 dilution in DCFH solution), a pre-used functioning heating element was installed into the refillable ENDS. In the first two trials, aerosols produced by e-liquid filled into the clearomizer resulted in detection of OX/ROS and the DCF fluorescence values attained did not require 1:10 dilutions (**Table 3, Experiment 3**). In contrast, aerosols produced in trials 3 and 4 were carried out by “dripping” small amounts of e-liquid sufficient to absorb into the wick without any liquid placed into the clearomizer. Aerosols produced in this manner resulted in high range DCF fluorescence values which required 1:10 dilutions in DCFH solution to attain fluorometer readings (**Table 3, Experiment 3**). These results suggest that the emerging trend of “dripping” e-liquids to produce ENDS aerosols delivers a larger dose of OX/ROS to consumers.

### Reactivity of commercial e-fluids with DCFH

**Fig 3:**
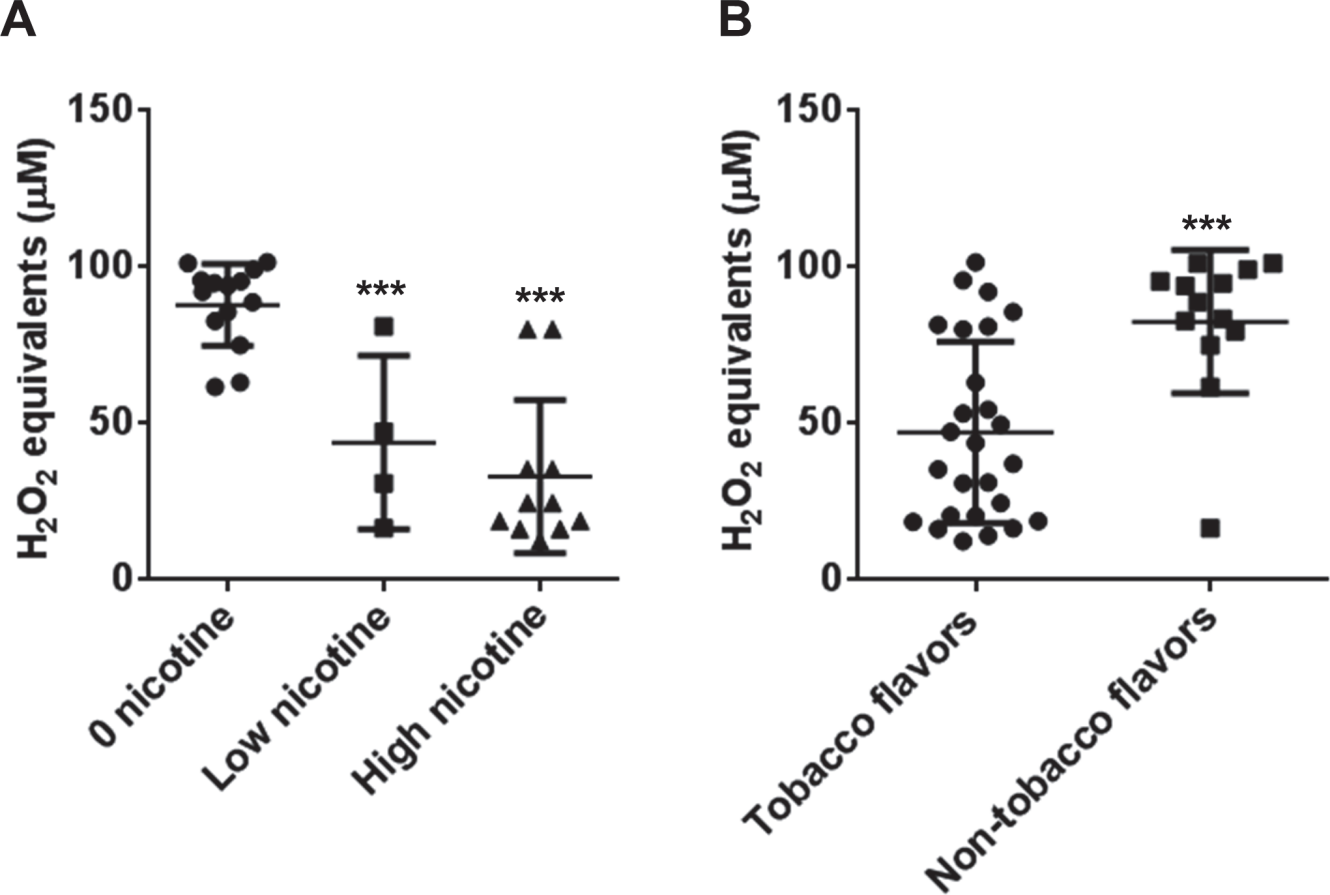
E-liquid reactivity with DCFH exhibits differences between nicotine content and flavor additives. (A) Commercially available e-liquids with different nicotine content. No nicotine (0 mg), low nicotine (6–12 mg) and high nicotine (16–24 mg). Data are shown as mean ± SD. *** P < 0.001. (B) Comparison of commercially available e-liquids, tobacco flavors (Tobacco, American tobacco, Classic tobacco 9x Tobacco, Marbo) versus non-tobacco flavors (Very berry, AMP, Mountain dew, Cinnamon roll, Grape vape, Cotton candy, Strawberry zing, Strawberry fields, Peaches n cream, Berry intense, Pineapple express, Melon mania, and Coconut). Data are shown as mean ± SD of n = 3, *** P < 0.001. Y-axis equal to DCF fluorescence Intensity Units (FIU).

A variety of locally purchased commercially available e-liquids differing in nicotine content and or flavor were reacted with the DCFH solution directly. Water and the purified humectants propylene glycol and glycerin showed no appreciable indication of reactivity with DCFH. All of the flavored e-liquids exhibited various DCFH reactivity (**Table 4)**. When e-liquid DCF fluorescence values from Table 4 were compared by nicotine content irrespective of brand or flavor, the nicotine containing e-liquids exhibited significantly less DCFH reactivity (**Fig. 3A**). Table 4 depicting e-liquids that contained non-tobacco flavor additives (dessert, fruit, and candy) where on average significantly more reactive with DCFH than e-liquids recreating tobacco flavors (**Fig. 3B**), suggesting more oxidative reactivity and injurious response by flavored e-liquids.

**Table 4:** DCF fluorescence of refillable e-liquids with different flavors and nicotine concentrations after addition of DCFH solution analyzed by a cell-free ROS assay.

**Vape drops** ^†^			**Vape dudes** ^†^		
Flavors	Nicotine conc. (mg)	DCF (FIU)	Flavors	Nicotine conc. (mg)	DCF (FIU)
Tobacco	0	63.15	Classic tobacco	0	95.82
Tobacco	6	30.92	Classic tobacco	24	54.48
Tobacco	11	16.47	Cinnamon roll	0	82.8
Tobacco	18	16.33	Grape vape	0	75
Tobacco	24	18.64	Cotton candy	0	94.08
Very berry	0	101.3	Strawberry zing	0	101.3
AMP	0	83.53	Strawberry fields	0	61.71
**Ecto** ^†^			**Drip** ^†^		
American tobacco	0	85.69	Peaches’n cream	0	95.52
American tobacco	12	81.07	Berry intense	0	88.87
American tobacco	18	80.2	Pineapple express	0	99.28
American tobacco	24	81.65	Melon mania	0	94.94
**Upstate vape** ^†^			**Encore** ^†^		
9x Tobacco	0	92.2	Tobacco	16	12.43
9x Tobacco	11	47.4	Tobacco	24	14.16
9x Tobacco	18	35.4	**Roc juice** ^†^		
9x Tobacco	24	31.21	Tobacco	0	101.2
Marbo	0	43.79	Tobacco	6	20.6
Marbo	6	49.71	Tobacco	18	18.79
Marbo	11	37.14	Tobacco	24	20.52
Marbo	18	24.57	Coconut	24	16.62
Marbo	24	53.32			
Mountain dew	18	79.62			

^†^ 167 μl e-liquid added to final volume of 5 ml DCFH solution, equivalent to volume of H_2_O_2_ added to attain a fluorometric value of approximately 50 FIU. DCF fluorescence for 50 μM H_2_O_2_ standard (50.02), humectant polyethylene glycol (0.43), humectant glycerin (1.3) and vehicle/water (0.03).

### Human lung fibroblasts exhibited stress and morphological change in response to e-liquids/humectants

**Fig 4:**
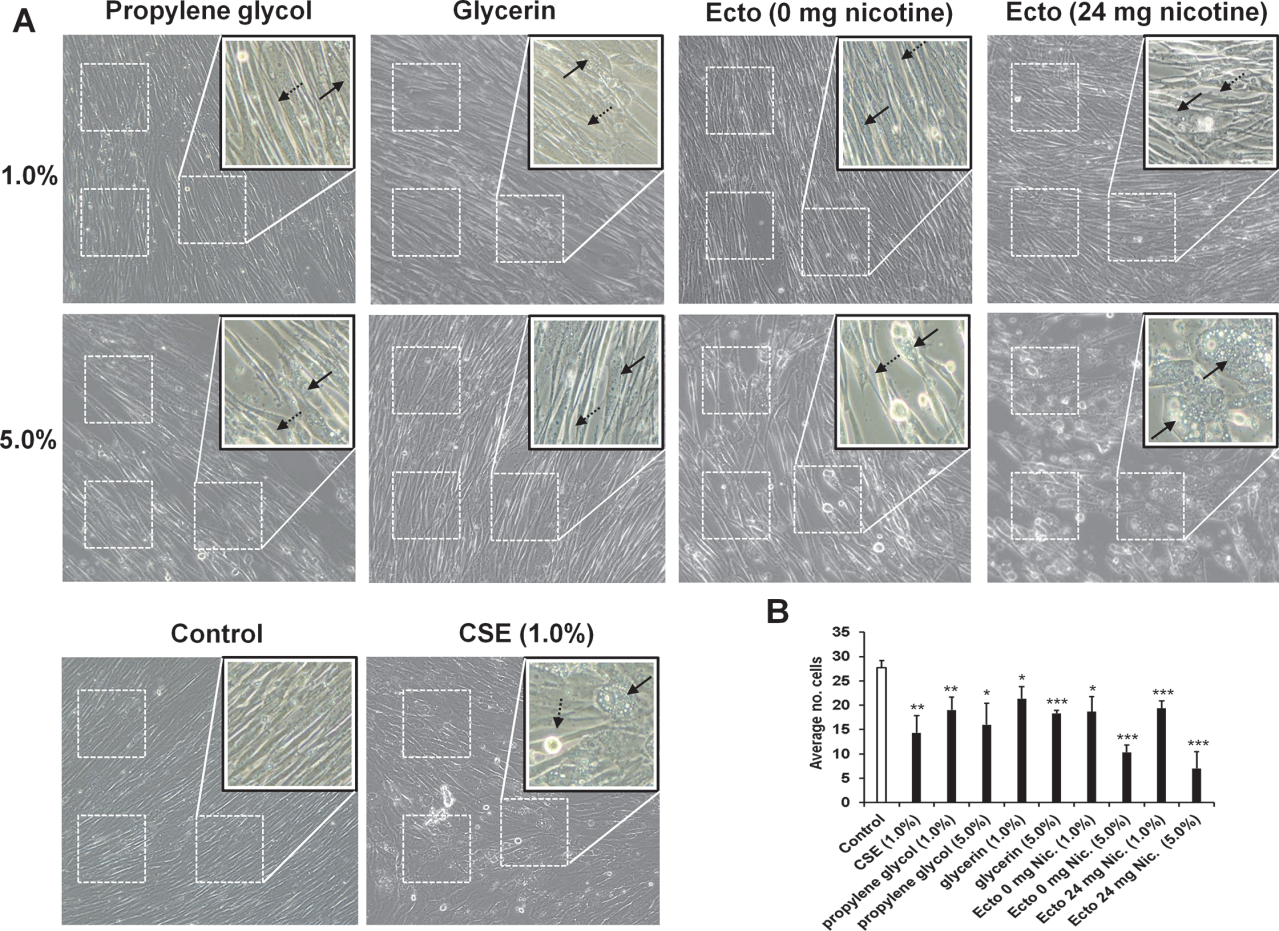
Addition of e-liquids to cell culture media induces morphological changes in human lung fibroblasts. (A) HFL-1 cells grown to 95% confluence were treated with the following e-liquids; propylene glycol, glycerin, or Ecto American tobacco flavor for 24 hrs and then examined for morphological changes by phase-contrast microscopy. Treatment of HFL-1 with 1.0% CSE for 24 hrs included for comparison. Images captured at 20x magnification. Embedded images show expansion of defined area of monolayer as demarcated by dashed boxes. Representative images are shown (n = 3). Enlarged vacuolarized cells in expansion area or large circularized cells (solid arrow), and areas of lost cell-cell connection next to spindle formations (dashed arrow) within defined area vs control. (B) Average number of cells counted adjacently across a single diagonal of 3 defined areas placed randomly (dashed boxes within images). The direction of diagonal cell counts is based on cell orientation in each image. Data are shown as mean ± SD. *P < 0.05; **P < 0.01; and *** P < 0.001 as compared to untreated control culture in growth media.

The effect of exposing lung cells directly to e-liquids is not known. Since OX/ROS reactivity is associated with ENDS e-liquids in the cell-free conditions, we asked whether or not commercially available e-liquids or purified humectants may induce any obvious morphological signs of cell stress in normal human primary lung cells. After 24 hrs, near to confluent fibroblasts treated in either 1% or 5% propylene glycol, glycerin, or tobacco flavored commercial e-liquid (Ecto) exhibited various morphological alterations (**Fig. 4A**). Fibroblasts cultured with e-liquid or CSE exhibited a reduction in the number of cells per count area (**Fig. 4B**). Many of the treated cells were enlarged and vacuolarized, and this effect was greater in CSE treated cells and cells treated with 5% e-liquids (**Fig. 4A)**. Compared to control cells, e-liquid and CSE treated cells showed hetero-morphological structures (enlarged cells and spindle formation) in e-liquid treated cells. Commercially available e-liquid added to cells at 1% concentration without nicotine, displayed similar morphological alterations to that of 1% propylene glycol. In contrast, fibroblasts cultured in 1% e-liquid that do contain nicotine, resulted in more profound morphological changes that resemble cells treated with 1% CSE [37]. There was also considerable cell overlap and mixed directional orientation throughout the image field for cells treated with 1% e-liquid containing nicotine. Vacuolization and cell enlargement following treatment with 5% e-liquid containing nicotine was most similar to fibroblasts treated with 1% CSE. There is also almost complete loss of the fusiform structure typical of fibroblasts in culture (control). Both CSE and e-liquid treated cells also showed a prevalence of larger adhered circular cells, noticeable due to the halo effect inherent in phase-contrast microscopy. These results suggest when e-liquids are applied directly to lung fibroblasts at these concentrations, there were signs of cell stress and other phenotypic abnormalities that are further exacerbated by nicotine.

### ENDS e-liquids/humectants and cell viability

To assess how e-liquids/humectants affect cell viability relative to CSE treatment, normal human lung fibroblasts were first cultured in 35 mm dishes and grown to 90% confluence. The cells were then shifted to medium containing 2.5% propylene glycol, glycerin, commercial e-liquids (0 mg or 24 mg of nicotine), or CSE (1%) and measured for viability after 24 hrs. A treatment condition of 1% CSE was also included, in which we have been able to maintain high density normal human lung fibroblasts for over 24 hrs without a significant cell loss/viability [38].

Lung fibroblast viability following treatments with 2.5% propylene glycol, glycerin, or commercial e-liquids was not significantly different than control after 24 hrs (Control; 90.53 ± 5.34, Propylene Glycol; 88.40 ± 2.99, Glycerin; 91.97 ± 6.23, Ecto American tobacco flavor 0 mg nicotine; 92.7 ± 2.55, Ecto American tobacco flavor 24 mg nicotine; 78.57 ± 6.67, % viability in means ± SD, p > 0.05%). As expected, 2.5% CSE treatment caused significant cell death, leading to less than 20% viability after the 24 hrs (CSE; 12.7 ± 4.73, % viability in means ± SD, p < 0.001). Conversely, viability for fibroblasts treated with 1% CSE was not significantly different than control, similarly to cell treatments with 2.5% e-liquid/humectant (CSE; 89.4 ± 5.86% viability in means ± SD). Therefore, the lung fibroblasts were more sensitive to CSE than e-liquids/humectants which did not exhibit an apparent effect on cell viability when treated at a higher concentration than CSE. However, in our initial assessments, we observed a global decrease in HFL-1 cell viability when the cells were cultured within smaller growth areas (15.5 mm dishes). E-liquid concentrations starting at 0.5% decreased cell viability below 50% for three of the commercial e-liquid brands after 24 hrs (**Table 5**). The decrease in cell viability broadened after increasing the e-liquid concentration to 5% and then 10% in the smaller culture dishes. Since cell viability after treatment with 2.5% e-liquid/humectant in larger culture areas was not reduced compared to control cells, the susceptibility to loss of cell viability by direct addition of e-liquids to culture media depends on size of the cell population.

**Table 5:** Effect of e-liquids on HFL-1 cell viability in small 24-well culture area after 24 hours.

E-liquid (%)	Nicotine concentration (mg)											
	0			12			18			24		
	UV	E	RJ	UV	E	RJ	UV	E	RJ	UV	E	RJ
0.5	85.9	70.2	40.5^Ŧ^	47.9^Ŧ^	31.3^Ŧ^	26.5^Ŧ^	82.9	89.4	24.6^Ŧ^	58.5	68.5	19.0^Ŧ^
5.0	42.6	33.0	16.4	21.5	54.0	29.7	25.0	36.8	18.0	16.5	10.9	13.1
10.0	20.7	1.3	27.1	1.4	0.0	36.4	1.2	0.8	1.0	0.0	0.0	0.0
Mean ± SD												
0.5	65.5 ± 23.05			35.2 ± 11.2*			65.6 ± 35.7*			48.7 ± 26.2*		
5.0	30.7 ± 13.3			35.1 ± 16.9*			26.6 ± 9.5*			13.5 ± 2.82*		
10.0	16.4 ± 13.4			12.6 ± 20.6*			13.5 ± 2.8*			0.0 ± 0.0*		

HFL-1 control cells without any treatment showed 95.4% viability.

E-liquids (UV: Upstate vape; E: Ecto; and RJ: Roc juice) at concentrations were used in this study (0.5%, 5.0% and 10.0%) for measuring percentage viability in HFL-1 cells after 24 hrs treatment.

^Ŧ^ values below 50% viability in 0.5% e-Liquid.

* NS compared to 0 mg nicotine

### ENDS e-liquid flavor additives mediate release of IL-8 in lung fibroblasts

**Fig 5:**
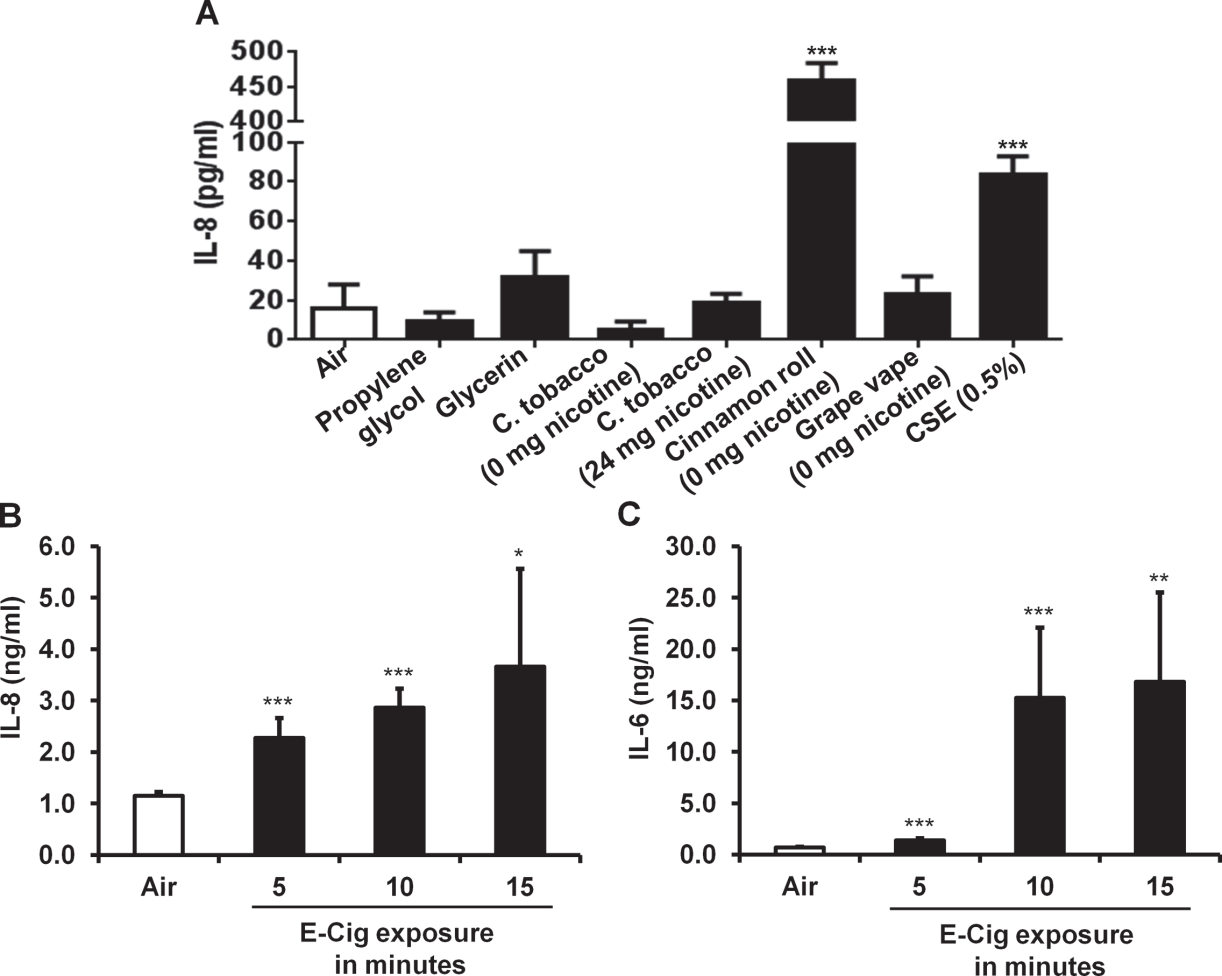
Inflammatory mediators secreted by human lung fibroblasts (HFL-1) treated with e-liquids/humectants and human epithelial airway cells (H292) treated by air-liquid interface with e-cigarette aerosols. (A) Levels of IL-8 release in conditioned media from HFL-1 cells treated for 24 hrs with 1% humectants or e-liquids or CSE were measured by ELISA. Data are shown as mean ± SD of n = 3. *** P < 0.001 compared to control cells maintained in media with 0.5% FBS. (B) H292 cells were exposed to Blu e-cigarette aerosols with a puff of 3–4 sec for 5, 10 and 15 min. After exposure, H292 cells were incubated at 37°C in 5% CO_2_ incubator for 16 hrs and levels of IL-8, and (C) IL-6 release in conditioned media were measured by ELISA. Data are shown as mean ± SD. *P < 0.05; **P < 0.01; and *** P < 0.001 as compared to air group (cells maintained in incubator).

Interleukin 8, a cytokine that functions as a chemoattractant for inflammatory leukocytes and is released from lung cells after exposure to cigarette smoke was measured in conditioned media from normal human lung fibroblasts treated with 1% e-liquids/humectants or 0.5% CSE. Neither of the pure humectants (propylene glycol, glycerin) elicited significant increase in release of IL-8 compared to control group (15.9 ± 12.02 pg/ml) after 24 hour treatment (**Fig. 5A**). Of the four commercially available e-liquids (Vape Dudes), only cinnamon roll flavored e-liquid stimulated a significant increase in IL-8 secretion (458.14 ± 26.20 pg/ml). The tobacco flavored e-liquid containing 24 mg nicotine, although eliciting slightly higher levels of IL-8 secretion than control cells (18.60 ± 4.79 pg/ml), was not statistically significant. However, although IL-8 secretion by tobacco flavored e-liquid containing 0 mg of nicotine was also not significantly different from the control group, the tobacco flavored e-liquid containing 24 mg of nicotine yielded significantly higher IL-8 levels (18.59 ± 4.79 pg/ml) compared to tobacco flavored e-liquid containing 0 mg of nicotine (5.28 ± 4.03 pg/ml). This suggests that nicotine added to e-liquid had a striking effect on IL-8 secretion in lung fibroblasts. Treating cells with 0.5% CSE, significantly increased fibroblast IL-8 secretion (83.81 ± 8.99 pg/ml). Since cinnamon flavor e-liquid is capable of stimulating a significant increase in IL-8 secretion from lung fibroblasts, while other e-liquid flavors (tobacco and grape) do not, certain e-liquid flavor additives can stimulate an inflammatory response in cultured lung fibroblasts.

### Human airway epithelial cells directly exposed to e-cigarettes vapor increase IL-8 and IL-6 secretion

Using an air-liquid interface culture system, human lung H292 epithelial cells were directly exposed to tobacco flavor Blu e-cig aerosols. IL-8 and IL-6 secretion measured at 16 hrs after air-liquid interface exposure for each exposure time period was significantly higher than air groups (**Fig. 5B, D**). The release of IL-6 into culture media also occurred in a dose-dependent manner in response to the aerosol exposures. IL-6 secreted following 10 minute exposures to e-cig aerosols were significantly higher than the 5 minute exposures (**Fig. 5C**). The IL-8 levels induced by air-liquid interface aerosols in H292 were all significantly increased compared to air group. However, they did not exhibit a dose-dependent effect over increasing exposure periods (**Fig. 5B**).

**Fig 6:**
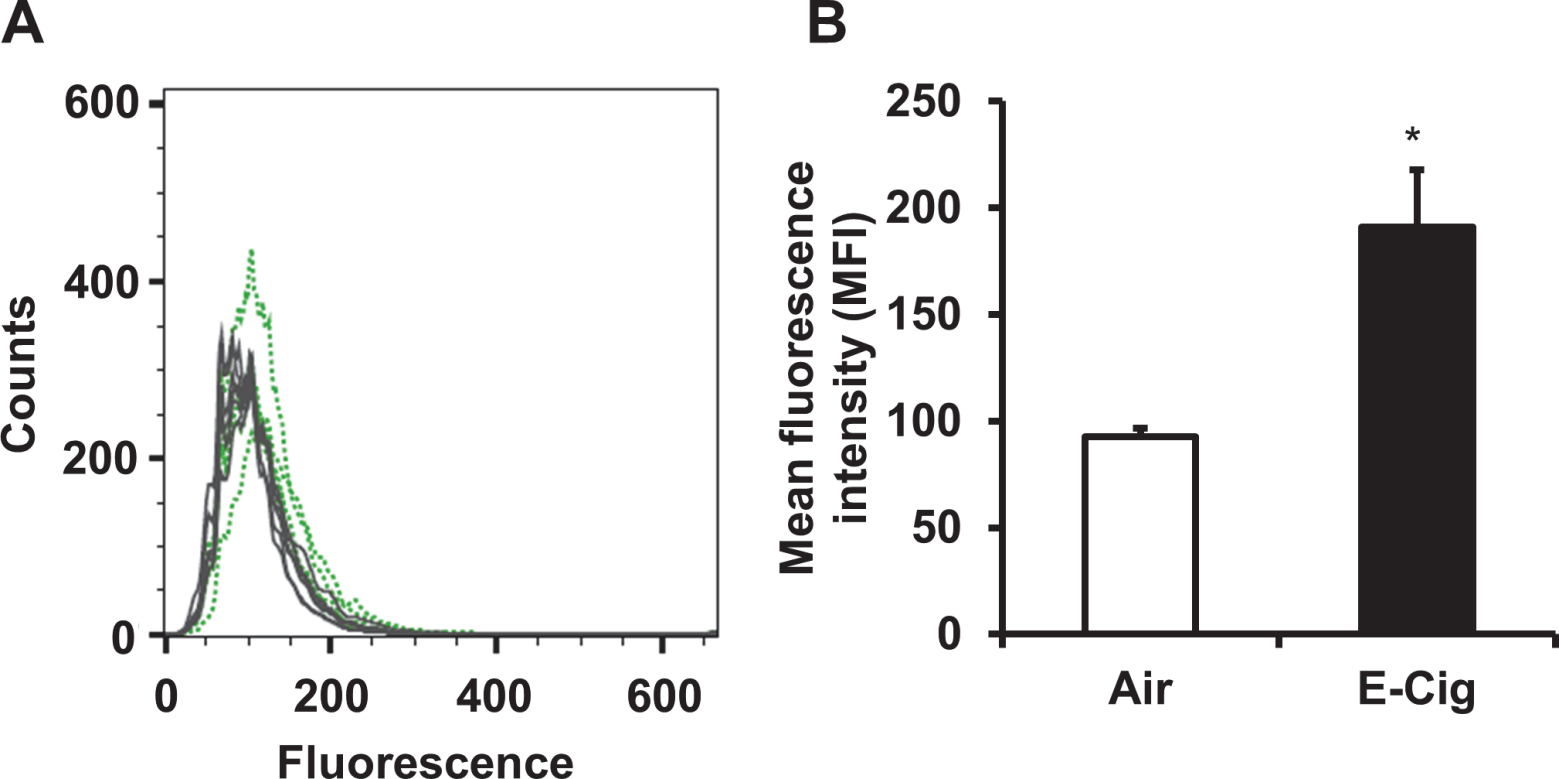
Air-liquid interface deposition of fluorescent substance on human bronchial airway epithelial cells. Beas-2B cells exposed to Blu e-cigarette vapor with a puff of 3–4 sec for 15 min. After exposure cells were immediately collected and measured by flow cytometry. (A) Histogram showing increase in non-specific fluorescence in cells exposed to e-cig aerosols. (B) Average fluorescence for e-cig exposed cells versus air-sham control shown as Mean Fluorescence Intensity (MFI). Data are shown as mean ± SD, n = 3, * P< 0.05 compared to air-sham control cells.

Next, we observed evidence of a non-specific e-cig substance associated with its aerosol that could emit a fluorescent signature after aerosol deposition onto the cells in the air-liquid interface chamber that may be associated with oxidative and inflammatory responses. Beas-2B cells exposed for a 15 minute period (4 sec. puffs every 30 sec.) with Blu e-cig aerosols were harvested and analyzed by flow cytometry using 2 different colored lasers (488, and 405 nm). In cells exposed to e-cig aerosols, we detected a small but significant increase in fluorescence utilizing the 405 nm laser with 440/40 band pass filter (**Fig. 6**). This result alludes to the possibility that e-cig aerosol constituents can adhere to cell surfaces despite those surfaces being submerged under a thin layer (1–2 mm) of culture media, and become pro-oxidant and inflammatory.

### E-cigarette aerosol exposure in mice caused lung inflammation and pro-inflammatory response

**Fig 7:**
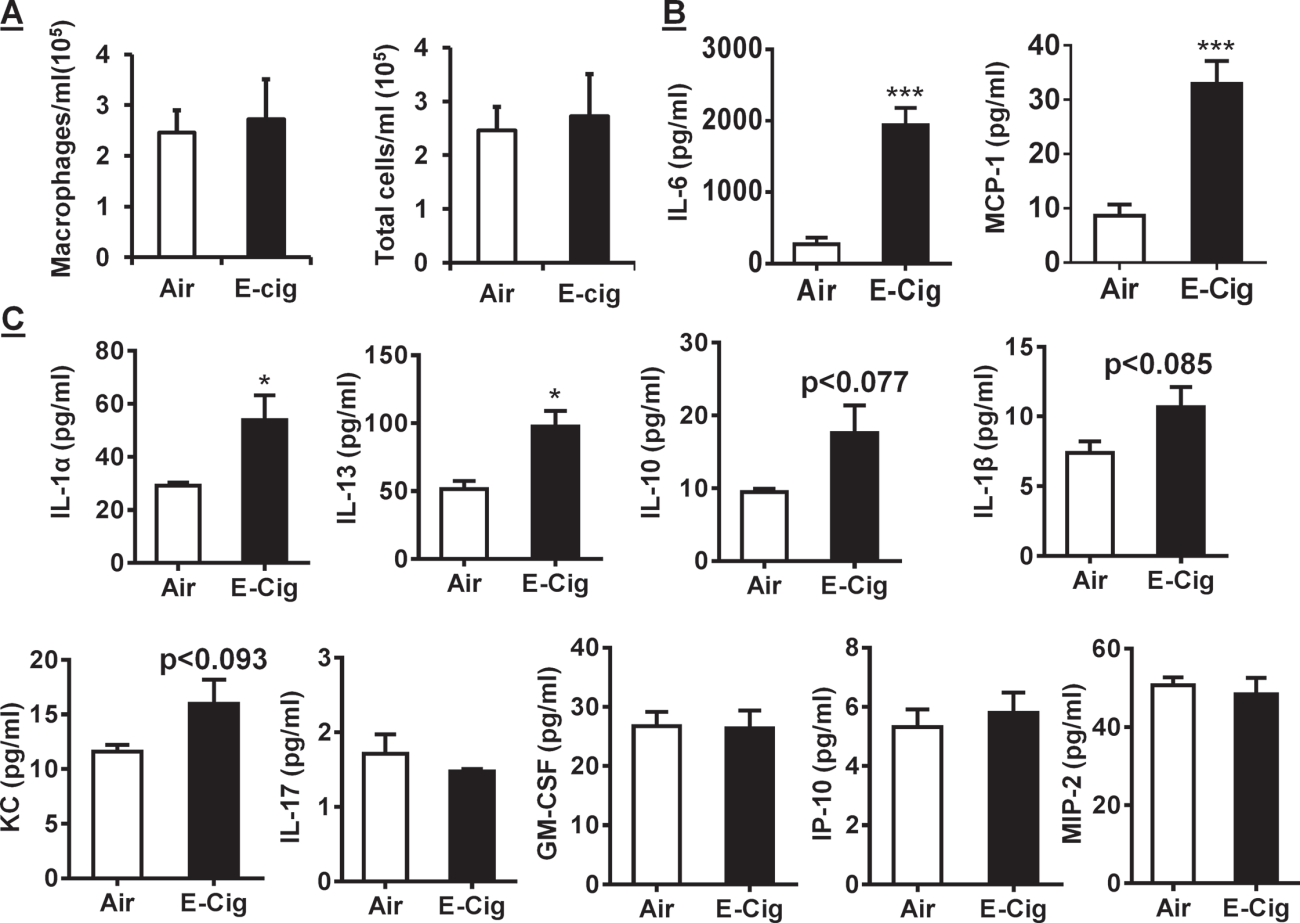
Acute e-cigarette aerosol exposure causes lung inflammation and pro-inflammatory response in mouse lungs. WT Mice (C57BL/6J) were exposed to e-cigarette aerosol exposure (200 mg/m^3^ TPM) for 3 days and sacrificed 24 hrs after the last exposure. (A) At least 500 cells in the bronchoalveolar lavage fluid (BALF) were counted with hemocytometer to determine the number of macrophages and total cells on cytospin slides stained with Diff-Quik. (B) Levels of pro-inflammatory mediators MCP-1 and IL-6 were measured in BAL fluid obtained from room air and e-cig aerosol exposed mice (C57BL/6J). Data are shown as mean ± SD. *** P < 0.001 compared to air group mice (C) Cytokine/chemokine levels in BAL fluid from room air and e-cig aerosol exposed mice were also measure using Luminex multiplex assay. Data are shown as mean ± SEM, n = 3, *P < 0.05 compared to air group mice (C57BL/6J).

C57BL/6J mice were exposed to side-stream Classic tobacco flavor (16 mg nicotine) e-cig aerosols for 3 days (acute exposure). On average, macrophage counts were higher in e-cigs exposed mice, but were not statistically different compared to air group controls (**Fig. 7A**). The total cell counts in BAL fluid 24 hrs after the last exposure exhibited higher average numbers of cells, yet were not significant compared to air group controls (**Fig. 7B**). Analysis of BAL fluid collected 24 hrs after the last exposure (3^rd^ day) to aerosols demonstrated pulmonary inflammation. MCP-1, a potent macrophage chemotactic cytokine was significantly increased in e-cigs aerosol exposed mice compared to air group controls (**Fig. 7B**). Levels of IL-6 which modulates a number of immune-inflammatory pathways in target leukocytes is significantly increased in BAL fluid from e-cigs exposed mice compared to air group controls (**Fig. 7B**).

To further assess the inflammatory response to side-stream e-cigs aerosol in mouse lung, a panel of cytokines/chemokines in BALF was measured in room air and e-cigs aerosol exposed mice using a Luminex kit (see Materials and Methods). Levels of IL-1α and IL-13 were significantly increased in e-cigs aerosol exposed mice compared to air group controls (**Fig. 7C**). Levels of IL-17, GM-CSF, IP-10, and MIP-2 in BALF did not change in response to e-cig side-stream aerosol. Levels of IL-1α, IL-1β, and IL-13 were slightly increased in e-cig aerosol exposed mice but not significant compared to air group controls. These data indicate that acute side-stream exposure to e-cig aerosol in mouse lung is sufficient to elicit an inflammatory response due to increased levels of proinflammatory mediators.

### Plasma cotinine levels in e-cig vapor exposed mice

Blood collected from mice sacrificed immediately after acute Blu e-cig exposure (3^rd^ day after 5 hrs exposure) was used to measure cotinine levels, a nicotine metabolite [39]. Plasma cotinine levels reached an average of 10.78 ± 7.80 ng/ml for e-cig exposed mice. The plasma from mice sacrificed 24 hrs after the last exposure did not show any detectable cotinine levels (ND) after e-cigs exposure.

### Intracellular glutathione levels in mouse lung exposed to short-term chronic e-cigarette aerosols

**Fig 8:**
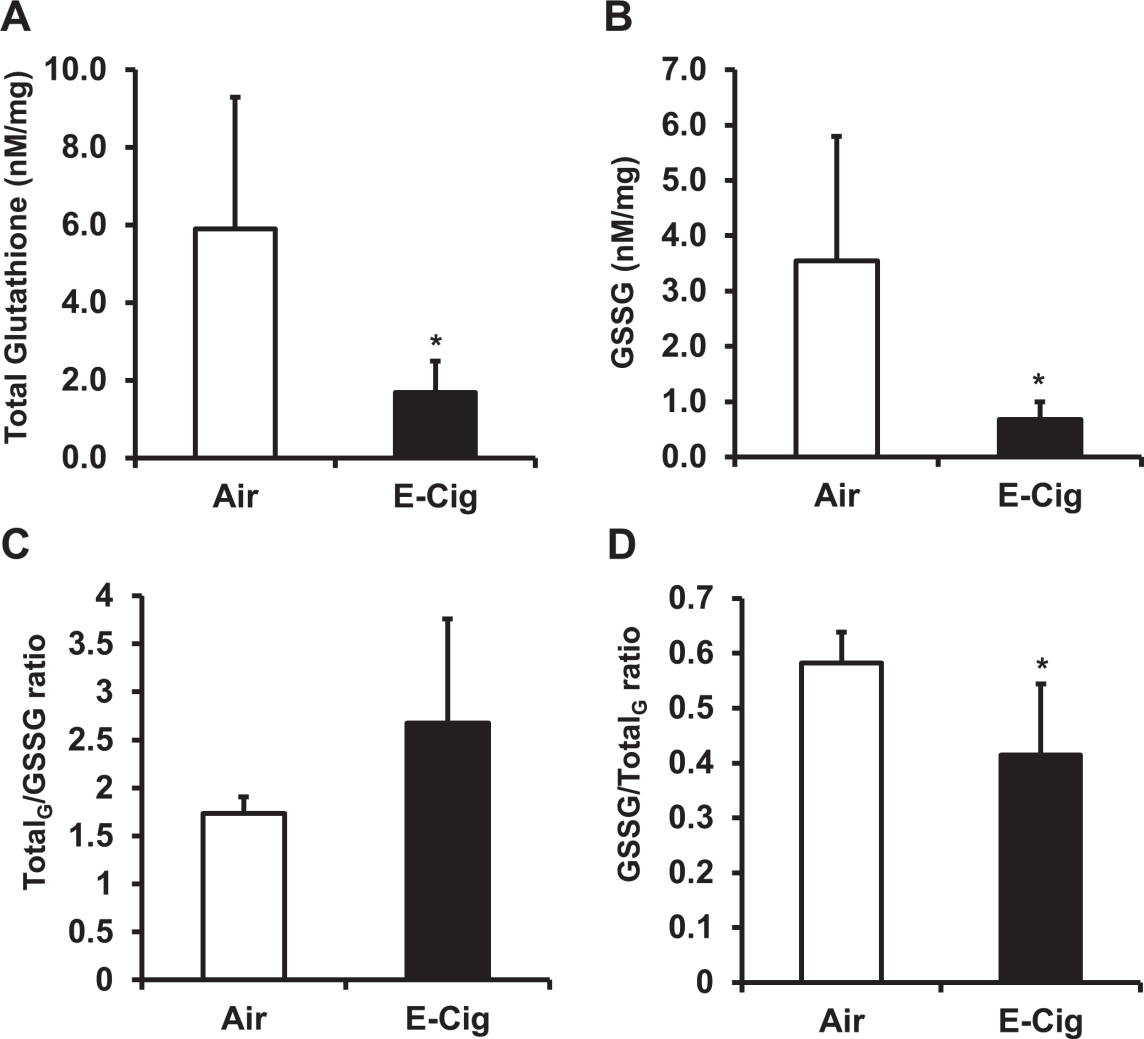
Intracellular glutathione levels in mouse lung following acute e-cigarette aerosol exposure. Mice were exposed to e-cig aerosol exposure (200 mg/m^3^ TPM) for 3 days and sacrificed immediately after the last exposure (3^rd^ day after 5 hrs exposure). Levels of (A) Total glutathione. (B) glutathione disulfide GSSG. (C) Total glutathione to GSSG ratio and (D) GSSG to total glutathione ratio were measured in lung homogenates. Data are shown as mean ± SD (n = 3/group).* P < 0.05 compared to air group mice (C57BL/6J).

Both total and oxidized forms of glutathione were assessed. Glutathione levels in mouse lung lysates following animal exposure to side-stream Blu e-cig aerosols were depleted. Air group levels of glutathione averaged 5.89 ± 3.40 nM/mg protein while the average glutathione level for animals exposed to e-cig aerosols was reduced to 1.69 ± 8.10 nM/mg protein (**Fig. 8A**). Oxidized (glutathione disulfide) levels of glutathione (GSSG) for air group averaged 3.54 ± 2.25 nM/mg protein and were decreased to 0.68 ± 0.32 nM/mg protein in exposed animals (**Fig. 8B**).

Ratios of total Glutathione to GSSG and *vice versa* were measured to determine if there was an effect from the side-stream e-cig aerosols on the balance between reduced and oxidized forms of glutathione within the lung. The ratio for total glutathione to GSSG was not significantly different between Air group and e-cig exposed animals (**Fig. 8C**). There was however, a small decrease in the ratio for GSSG to total glutathione (**Fig. 8D)**. These results suggests that total glutathione levels are reduced by e-cig aerosols and the redox balance between the reduced and oxidized forms of glutathione is affected by side-stream e-cig aerosol inhalation as well.

## Discussion

ENDS/e-cigs have become prominent fixture in the consumer landscape. Habitually inhaling their aerosols has been implicated by manufacturers as a safer alternative to smoking conventional cigarettes and many electronic cigarette users have adopted similar perspectives [40]. However, recent e-cigs studies showing that there are substantial levels of nanoscale particles in addition to detectable levels of metals with toxic materials (e.g., aluminum, copper, magnesium, zinc, lead, chromium, manganese, and nickel) in e-cig aerosols brings this view into question [10]. At the nanoscale size, particles may reach the alveolar epithelium and mediate oxidative stress and inflammation [41,42].

It is not yet certain what the exact factors are associated with ENDS that might mediate oxidative stress. The eGO Vision ENDS vaporizer with refillable chamber and exchangeable heating element were employed to detect reactive OX/ROS under a variable set of parameters, such as the ratios of pure humectant mixtures, commercially available e-liquids, changeable voltage settings, and state of heating element. The ability to manipulate these parameters individually facilitates determination of the OX/ROS source from the vaporizer.

Using the above parameters, our results indicate that there are a number of variables that affect OX/ROS production in ENDS/e-cigs that are not exclusive to e-liquid aerosols. For example, it was observed that OX/ROS reactivity in aerosols produced from the refillable ENDS device varied between relatively high or low levels. For instance, in some cases DCF fluorescence values from refillable ENDS aerosols approached or overlapped air-sham control values despite aerosols being sufficiently produced during experimental trials that included vaporization of e-liquids/humectants. Multiple batches of DCFH solution prepared for additional experimental replicates may account for some of the variability seen for OX/ROS reactivity. The attainment of a number of unusually high fluorescence DCF measurements prompted us to question if the state of the heating element also influenced OX/ROS release from the device. We also noticed that each time a new heating element was installed into the eGo ENDS, a small amount of aerosol could be produced without addition of any e-liquid suggesting there may be volatile substances associated with ENDS heating elements following manufacturing.

The trend called “dripping” is intended to allow the user to achieve stronger ‘hits’ and also gives the option to more easily switch between flavors, brands, or nicotine content without frequently emptying and refilling the clearomizer chamber (communication with Mr. Douglas Done University of Rochester Department of Public Health Sciences) [43,44]. The design of refillable ENDS is suggested to incorporate “dripping” as an option for consumers [45]. The spike in OX/ROS release that resulted in high range DCF fluorescence after dripping e-liquid onto the 4^th^ use heating element wick led us to hypothesize that “dripping” rather than filling the clearomizer with e-liquid, which completely submerges the heating element, is potentially more hazardous. Our results indicate that the dripping method for ENDS usage is likely to generate a larger amount of OX/ROS.

As Goniewicz *et al* reports, heating e-liquids with sufficient temperatures produces detectible levels of formaldehyde, acrolein, and acetaldehyde carbonyls with the possibility that these compounds form due to the pyrolysis of glycerin [5,6]. However, in comparison to conventional cigarette smoke, levels of these carbonyls were found to be between 9 and 807 times lower suggesting that ENDS/e-cigs may be a safer alternative to conventional cigarettes [5,6]. Carbonyl levels in ENDS/e-cigs also appeared to depend on the brand of the device while the black deposits that we see from the heating elements following our experiments are consistent with what has been found associated with other devices [10,46]. Therefore, although toxic carbonyl by-products measured in ENDS aerosols may be orders of magnitude lower than conventional cigarettes as reported by Goniewicz *et al*. and Kosmider *et al*., the potential for delivering oxidizing agents as measured here may be currently underappreciated. The OX/ROS produced by “dripping” techniques that we observe coincides with emerging “vaping” trends that may place consumers at greater risk for lung damage. The higher volume of liquid surrounding the heating element when liquid is filled into the clearomizer during vaporization may implement an important cooling effect that prevents the device from producing temperatures high enough to form higher levels of combustion products or might mitigate the amount of OX/ROS released.

The nicotine containing e-liquids exhibited less reactivity with DCFH potentially due to low OX/ROS properties of nicotine in an aqueous solution [47]. Although comparison of the 0 mg and 24 mg nicotine Vape Dudes e-liquid aerosols produced by the refillable ENDS did not appear to be statistically different in DCF fluorescence to one another (large sampling error), the aerosols produced from the nicotine containing e-liquid was on average less than the samples without nicotine. This supports a possible trend in the reduction of OX/ROS in the presence of nicotine because the aerosols produced from the Blu e-cig cartomizer containing 16 mg nicotine exhibits a significant reduction in OX/ROS compared to the nicotine free cartomizer. Similarly, we observed unvaporized nicotine containing e-liquid was less oxidative to DCFH. Therefore, nicotine vaporized using either device, at least does not appear to contribute to OX/ROS generated.

E-liquid added directly to lung cells affects cell morphology, induces a stress phenotype and contributes to inflammatory response in a manner dependent on nicotine content and flavor choice. In this study, cinnamon flavored e-liquid elicited a strong IL-8 response compared to CSE which is consistent with the ability of cinnamon flavored e-liquids to induce cellular toxicity [2,3,48]. When cells are treated with nicotine containing e-liquid, filopodia appear to shorten similar to loss of filopodia in periodontal ligament gingiva fibroblasts treated with nicotine [49]. The accumulation of vacuoles we observe in cells treated with nicotine e-liquid is corroborated as well in other cells [37,49]. Pure propylene glycol and glycerin did not appreciably react with DCFH. However, cell morphology is affected by these humectants when cells were treated with them at the concentrations used in this study. The tobacco flavoring and possibly other additives exacerbated cell stress (enlargement, appearance of vacuoles). Propylene glycol and glycerin used in are FDA approved for use in foods, cosmetics, tobacco products, and is Generally Recognized as Safe (GRAS) [50]. However, direct exposure of these humectants to lung tissue and the concentrations that may accumulate in the lung from chronic ENDS use is not known. Propylene glycol may enhance the delivery of e-liquid additives of nicotine, flavors, and potential toxic impurities into the lung tissue. As skin penetration enhancer, propylene glycol is a mode of choice for transdermal drug delivery [51].

A limited number of studies have assessed the effect of commercially available e-liquids on cell toxicity and viability and attribute most of the toxicity being due to flavor additives [2]. The lung fibroblasts we treated with e-liquids showed no significant decrease in cell viability unless they were cultured in small wells with fewer numbers/density of cells. Mouse 3T3 fibroblast and rat myocardial cells treated with e-cig extracts were also minimally toxic and did not show appreciable effect on viability [3,52]. Other studies show cultured lung alveolar cells exposed to electronic cigarette aerosols rather than extract suggests that e-cigs induce toxicity in lung epithelial cells and lead to reduced viability in a manner that is dependent on flavor additive [53].

Epithelial airway H292 cells exposed to e-cig aerosols by air-liquid interface secrete proinflammatory cytokines, such as IL-6 and IL-8 into culture media after the cells were allowed to culture for a 16 hour response. Although, lower levels of particulate matter have been measured in e-cig aerosols compared to conventional cigarette smoke [54], it is not well understood how these particles affect inflammation in the lung. How ENDS/e-cigs compare to conventional cigarettes in mediating inflammatory responses will require further experiments in various settings, conditions, and cell lines to under the mechanisms.

Mouse exposure to e-cig aerosols was carried out with a modified a Teague smoke exposure machine. Generally this machine is employed for standardized small animal exposures to sidestream/second-hand smoke of conventional cigarettes [32,33]. BALF from wild type mice (C57BL/6J) at 24 hrs following exposure to short-term chronic Blu e-cig aerosols (3 days) showed inflammatory response as indicated by increased inflammatory mediators. Though we did not observe an appreciable difference in macrophage lung influx or altered levels of total cells in BALF from mice exposed to e-cig aerosols, cytokine MCP-1 which acts a macrophage chemokine was significantly elevated in mouse BALF. IL-6, which is a potent mediator of acute-phase inflammatory response, was also significantly elevated in BALF from mice exposed to e-cig aerosols. The small elevation in average macrophage and total cell levels in BALF after aerosol exposures, though not significantly different than ambient air-group, is in line with the increased levels of various cytokines we measured that have modulatory roles in immunity and inflammation.

The cotinine levels in e-cig exposed WT (C57BL/6J) mice fall within the same range as C57BL/6J mice exposed to side-stream cigarette smoke for 6 hours in addition to passive smoking by human non-smokers cohabitating with smokers [55,56]. Therefore, nicotine is indeed delivered into mouse blood using the Teague smoke exposure machine which is designed for passive second hand smoke. Inhalation of nicotine is sufficient to increase cotinine levels in the blood which has been associated with tobacco smoke induced emphysema in mice [57]. This highlights that ENDS may be harmful and injurious by chronic consumption.

Mouse exposure to conventional cigarette smoke for acute exposure has been shown to sufficiently diminish glutathione levels in the lung in a strain-dependent fashion [58]. Exposure of C57BL/6J mice to acute exposure of e-cig aerosols also decreased total and oxidized levels of lung glutathione. Altering the glutathione levels in lung cells through inhalation of e-cig aerosols could impose oxidative stress culminating in inflammatory response as seen by conventional cigarette smoke [1,25,26,38].

The Forum of International Respiratory Societies has recommended that ENDS/e-cigs sales be restricted until their safety is better evaluated due to the limited amount of information addressing health risks associated with ENDS/e-cigs use [59]. However, based on our data, the ENDS devices warrant regulation and not to be condoned as a method to transition away from conventional cigarette addiction. Nevertheless, further long-term/chronic studies are required to evaluate the health risks of ENDS/e-cigs.

In conclusion, we showed that 1) OX/ROS are generated by vaporizing ENDS/e-cig e-liquids/e-juices and are further influenced by the state of the heating element, 2) differences in OX/ROS reactivity in e-liquids prior to vaporization is associated with e-liquid flavor, 3) e-liquids can mediate effects on lung cell morphology and affect viability, 4) e-cig aerosols can modulate levels of oxidative stress and inflammation markers in both lung cells and mouse lungs, and 5) e-cig aerosols affect *in vivo* in lung glutathione redox physiology implicating oxidative stress. These data clearly demonstrate the lung toxicity and hazards of exposure to ENDS/e-cigarettes.
